# Trauma and orthopaedics training amid COVID-19: A medical student’s perspective

**DOI:** 10.1080/17453674.2020.1826658

**Published:** 2020-10-02

**Authors:** Carola Maria Bigogno, Kathrine S Rallis, Catrin Morgan, Rupen Dattani

**Affiliations:** Chelsea and Westminster NHS Foundation Trust, London, UK

*Sir,—*We read with interest the article by Dattani et al. (2020), describing the effects of coronavirus disease 2019 (COVID-19) on Trauma and Orthopaedics (T&O) training. As medical students of the “COVID Generation”, we take interest in this perspective. Yet, we notice there is little reflection on the pandemic’s impacts on undergraduate T&O education. Hence, we offer strategies to adapt undergraduate T&O teaching, highlighting telemedicine opportunities and limitations.

The COVID-19 pandemic has affected all fields worldwide, particularly healthcare training. Practical anatomy, clinical skills sessions, and hospital placements were suspended, raising concerns over medical students’ competency development. Restricted opportunities to practice physical examinations and observe clinical management (BOA 2014) has reduced students’ T&O specialty exposure, which can influence career selection (Johnson et al. 2012). Cancellation of undergraduate research placements, conferences, and electives has hindered learning, networking, and personal and professional development, while examination disruptions have prevented self-assessment. Although universities offered online resources, these are mainly limited to theoretical education, neglecting practical experience and simulation (Yaghobian et al. 2020). Moreover, although virtual patients are rated higher than recorded lectures (Courteille et al. 2018), these are underutilised and, still, cannot replace real-life clinical training. Dattani et al.’s telemedicine strategies for postgraduate trainees should be implemented more extensively in undergraduate medical education.

Telemedicine can enrich student education during COVID-19 and will likely continue being used, as the authors mention. Simulation and augmented reality are effective learning aids, especially for human anatomy assimilation (Moro et al. 2017). Simulation can prepare students for real-life T&O emergency situations, improving diagnostic reasoning, while reducing risks to patients (Lateef 2010). Small-group virtual teaching could maintain practical skill development which is essential for surgical T&O education. Encouraging students to observe and, when appropriate, participate in virtual clinics and surgeries would maintain access to healthcare environments, facilitating regular student-patient interaction whilst ensuring COVID-19 safety (Courteille et al. 2018, Stelian and Lacramioara 2018).

However, even though teleconsultations offer regular patient contact, it is harder to build rapport and trusted relationships with patients (Ekeand et al. 2010), and physical examinations are restricted (Romanick-Schmiedl and Raghu 2020). Moreover, with the expanding use of e-health there are concerns about quality of care and confidentiality of patient information, whist telemedicine can be technically challenging for patients (Romanick-Schmiedl and Raghu 2020). Hence, telemedicine may pose barriers for physicians, students, and patients.

It is therefore paramount to introduce teachings in medical school to familiarise students with telemedicine. Early training should teach students how to interact with patients through telecommunications, adapting communication skills to the online setting. Moreover, students should be learning how to triage patients that require hospital assessment and realise that telemedicine requires adaptation to the patient.

Medicine faces an unprecedented need for short- and long-term flexibility and adaptation. Doctors’ training must be adapted to minimise disruptions to career progression and quality of care. Equally, undergraduate medical education must be refined and reinvented to ensure future physicians’ competence and confidence in practice. Increasing understanding of telemedicine strengths and limitations is essential for physicians, students and patients.

**Carola Maria Bigogno ^1^ and Kathrine S Rallis ^1,2^**

*^1^ Barts and The London School of Medicine and Dentistry, Queen Mary University of London, London, UK*

*^2^ Barts Cancer Institute, Queen Mary University of London, London, UK*

*Email:* c.m.bigogno@se15.qmul.ac.uk

*Sir,—*Our article focused on the impact of the COVID-19 pandemic on postgraduate training within trauma and orthopaedics. However, as mentioned in the letter by Bigogno et al, it is also important to consider the impact of the pandemic on undergraduate education, as without medical students there is no next generation of orthopaedic surgeons. Thus, it is paramount to consider this perspective and to form strategies to help adapt undergraduate education in trauma and orthopaedics during the evolving COVID-19 pandemic.

As mentioned in the letter, the COVID-19 pandemic has caused large disruption to the undergraduate medical curriculum affecting medical students across all years, including many students in their clinical years being moved to the front line (Representatives of the STARSurg Collaborative, EuroSurg Collaborative, and TASMAN Collaborative. 2020). For many students, interest in a specialty begins in medical school and the medical school rotation experience has been shown to have an important role in shaping interest and perceptions within trauma and orthopaedics (Baldwin et al. 2011). It is vital that this undergraduate exposure to orthopaedics is not lost. The COVID-19 pandemic should be used as an unexpected opportunity for medical schools to review and improve their current curriculum content by adopting novel teaching methods to ensure the education of medical students’ is not compromised.

A lot of the strategies and concepts discussed in our article can be applied to both undergraduate and postgraduate education. The use of surgical simulation, virtual reality and telemedicine within the field of trauma and orthopaedics, is particularly relevant during the COVID-19 pandemic and medical schools should look at ways to integrate this further into the curriculum. The letter refers to the use of augmented reality as an effective learning aid for human anatomy (Moro et al. 2017). Mixed-reality headsets have also been used for ward rounds during the COVID-19 pandemic, helping reduce the number of people on the round and the amount of personal protective equipment used (Imperial College London. 2020). This reduces the time doctors and students spend in high-risk areas as well as providing an educational opportunity.

As orthopaedic surgeons, at all levels of training, it is our duty to work together with medical schools to help deliver a safe but effective undergraduate orthopaedic teaching rotation during the COVID-19 pandemic. As the possibility of a second wave approaches, medical student education should remain a priority.

Catrin Morgan and Rupen Dattani *Chelsea and Westminster NHS Foundation Trust, London, UK* *Email:*rupen.dattani@nhs.net
